# Characterization of C16–C36 alkane degradation and oily sludge bioremediation by *Rhodococcus erythropolis* XP

**DOI:** 10.1128/aem.02124-25

**Published:** 2025-12-03

**Authors:** Yan Zhang, Huan Liu, Shan Yu, Ruocheng Pei, Haiyang Hu, Weiwei Wang, Ping Xu, Hongzhi Tang

**Affiliations:** 1 State Key Laboratory of Microbial Metabolism, and School of Life Sciences and Biotechnology, Shanghai Jiao Tong University, Shanghai, People’s Republic of China; 2 Engineering Research Center of Agricultural Microbiology Technology, Ministry of Education & Heilongjiang Provincial Key Laboratory of Plant Genetic Engineering and Biological Fermentation Engineering for Cold Region and Key Laboratory of Microbiology, College of Heilongjiang Province & School of Life Sciences, Heilongjiang University 12432, Harbin, People’s Republic of China; Michigan State University, East Lansing, Michigan, USA

**Keywords:** *Rhodococcus erythropolis*, alkane degradation, oily sludge, bioremediation

## Abstract

Oil contamination poses significant risks to human health and ecosystems, emphasizing the importance of studying alkane biodegradation. In this study, we found that *Rhodococcus erythropolis* XP can utilize various alkanes, including C16–C36 *n-*alkanes and iso-alkane (pristane). The degradation capacity was significant, with over 95% of C20 degraded (500–2,500 mg/L) within 72 h. The bioremediation capacity in oily sludge was determined by a novel Low Pressure Gas Chromatography-Mass Spectrometry methodology especially for rapid analysis (within 12 min) of *n-*alkanes. Notable biodegradation of C14–C30 alkanes was observed in sludge treated with *Rhodococcus erythropolis* XP. In addition, metabolic intermediates of C16 and C20 were identified, indicating the presence of both terminal and subterminal pathways in *Rhodococcus erythropolis* XP. A new Baeyer-Villiger monooxygenase (BVMO_4041) was characterized, which catalyzes a key step in the subterminal pathway of alkane degradation. These results reflect the promise of *Rhodococcus erythropolis* XP in addressing the pressing need for efficient alkane degradation in contaminated environments.

**IMPORTANCE:**

Oil pollution posed a severe threat to human health and environmental safety due to its chemical stability and prolonged persistence. Although a lot of bacteria have been reported to degrade alkanes, the main components in oil pollution, it is urgent to identify strains that can degrade medium- and long-chain alkanes and to evaluate their performances during bioremediation. In this study, *Rhodococcus erythropolis* XP has been proved to obtain the almost strongest ability to degrade C16–C36 *n*-alkanes and branched alkanes (pristane), and to be a promising option for oily sludge bioremediation with newly developed rapid detection technology based on low pressure gas chromatography-mass spectrometry. Meanwhile, the metabolic pathways and a new *BVMO_4041* gene encoding Baeyer-Villiger monooxygenase were revealed. Our research provides a promising candidate for both practical bioremediation efforts and microbial research, and enriches the strain and gene resources for oil degradation.

## INTRODUCTION

Serving as a major energy source, petroleum has played an essential role in daily life and various industries, including manufacturing, chemicals, pharmaceuticals, etc. However, oil pollution due to the extraction process, transportation, and storage has posed a severe threat to human health and environmental safety (1, 2). The leaky oil can persist in the seawater for a long time, enhancing toxicity in fish and aquatic invertebrates (3, 4). Paraffin, saturated hydrocarbon (including straight and branched chains), is one of the main components in petroleum, classified into short- (<C8), medium- (C8–C18), and long-chain (>C18) alkanes (5). Compared to short-chain alkanes, medium- and long-chain alkanes obtain limited water solubility and high chemical stability. Indeed, their prolonged persistence in terrestrial and marine ecosystems has become an emerging concern. To address the challenges, bioremediation technology has proven to be an efficient, economical, and sustainable approach in recent years (6, 7).

During the past decades, a massive number of bacteria have been reported to utilize medium- and long-chain alkanes, including *Pseudomonas*, *Gordonia*, *Acinetobacter*, *Rhodococcus*, *Bacillus*, etc. (Table 1). For instance, *Pseudomonas aeruginosa* SJTD-1 and *Acinetobacter oleivorans* DR1 can both grow in the medium supplemental with C12–C30 *n-*alkanes, and *Dietzia* sp. DQ12-45-1b can utilize C6–C40 *n-*alkanes (8–10). However, most bacterial isolates exhibit limited consumption efficiency of medium- and long-chain alkanes. In 72 h, *Rhodococcus opacus* R7 can only metabolize 1 g/L C20 to 51% (11). It takes a week for *Acinetobacter pittii* SW-1 to utilize 500 mg/L of C18–C24 to over 80% and 5 weeks for *Bacillus* sp. NG80-2 to degrade 1% (wt/vol) C28 to 34.1% (12, 13). Moreover, very few strains, such as *Rhodococcus ruber* SBUG 82, can utilize branched-chain alkanes as substrates (14). Although the above strains have been partially applied to practical or simulated bioremediation, less is known about the change in microbial compositions during this process. Therefore, it is crucial to identify strains that can degrade contaminants efficiently and to evaluate their performances during bioremediation. Members of the *Rhodococcus* genus, found in many challenging locations, were recognized for their remarkable ability to survive and thrive under extreme temperatures, pH levels, and salt concentrations (15). Moreover, several *Rhodococcus* species exhibit an impressive ability to metabolize diverse xenobiotic compounds, such as polycyclic aromatic hydrocarbons (16). This property makes them promising candidates for bioremediation, given their capacity to degrade a wide range of alkanes.

**TABLE 1:** Bacterial strains capable of utilizing medium- and long-chain alkanes^*a*^

Strain	Carbon source utilization	Alkane degradation efficiency		Reference
		Substrate	Consumption rate	
*Acinetobacter pittii* SW-1	C18–C36 *n-*alkanes	500 mg/L of C18–C36 in 7 days	Over 80% of C18–C24, 30.29% and 13.37% of C32 and C36	(13)
*Acinetobacter nicotianae* KCC B35	C10–C40 *n-*alkanes	10 mg of C28 and C29 in heavy cell suspension during 24 h	32.0% of C28, 33.8% of C29	(17)
*Acinetobacter oleivorans* DR1	C12–C30 *n-*alkanes	N.M.	N.M.	(9)
*Acinetobacter venetianus* 6A2	C10–C40 *n-*alkanes	N.M.	N.M.	(18)
*Alcanivorax* sp. Est-02	C14–C28 *n-*alkanes	0.5% (wt/vol) mixture of C14–C28 in 14 days	60% of C24, 65% of C28	(19)
*Bacillus licheniformis* DM-1	C12–C36 *n-*alkanes	1% (wt/vol) C12–C36 in 10 days	Over 70% of C16–C22, 7.65% of C36	(20)
*Bacillus* sp. NG80-2	C15–C36 *n-*alkanes	1% (wt/vol) C15–C36 in 35 days	34.1% of C28	(12)
*Dietzia* sp. DQ12-45-1b	C6–C40 *n-*alkanes, cyclohexane, and isocetane	C14–C40: 0.30% (vol/vol) for liquid alkanes, and 0.05% (wt/vol) for solid alkanes in 21 days	13.87–121.41 mg/ (100 mL)	(10)
*Pseudomonas aeruginosa* SJTD-1	C12–C30 *n-*alkanes	500 mg/L of C14, C16, C18 in 36 h; 2 g/L of C16 in 72 h	Consumed	(8)
*Pseudomonas aeruginosa* GOM1	C8–C28 *n-*alkanes	0.5% light crude oil in 30 days	A total of 96% of C12–C38 *n-*alkanes	(21)
*Rhodococcus opacus* R7	C12–C24 *n-*alkanes	1 g/ L of C12–C24 in 72 h	88%, 69%, 51%, and 78% of C12, C16, C20, and C24	(11)
*Rhodococcus* sp. CH91	C16–C36 *n-*alkanes	0.2% C20–C36 in 21 days	Between 27.4% and 98.9%	(22)
*Rhodococcus ruber* SBUG 82 *Mycobacterium neoaurum* SBUG 109	Pristane, C16	0.01% (vol/vol) pristane in 8 h and 0.05% (wt/vol) of C20 in 21 days	93% pristane and 51% C20; 96% pristane and 59% C20	(14)
*Rhodococcus erythropolis* XP	Pristane, C16–C36 *n-*alkanes	2,500 mg/L of C20 in 72 h and 0.1% (vol/vol) pristane in 60 h	95% C20 and 80% pristane	This study

^*a*^ N.M., not mentioned.

Under toxic conditions, four pathways have been proposed in alkane degradation: the terminal, bi-terminal, subterminal, and Finnerty pathways (23). Most studies focused on the enzymes involved in the terminal oxidation pathway, e.g. alkane monooxygenase (AlkB), Cytochrome P450, and long-chain alkane monooxygenase (LadA); however, few of them elucidated the enzymes in the subterminal pathway (24). Baeyer-Villiger monooxygenase (BVMO) is an enzyme that can convert a ketone into an ester, participating in the subterminal pathway. More importantly, it functions as a significant biocatalyst in the synthesis of drugs and high-value chemicals. Understanding the substrate specificity of BVMO is essential for illustrating the physiological role of BVMO in alkane metabolism and its potential application in biocatalysis.

Gas chromatography-mass spectrometry (GC-MS) is a versatile and widely used analytical technique for organic compound analysis. Nonetheless, to detect substrates with high boiling points, especially long-chain alkanes, traditional GC-MS methods can be time-consuming (usually 20–40 min) because of a long-time gradient heating program. To overcome this disadvantage, Low Pressure Gas Chromatography-Mass Spectrometry (LPGC-MS) has recently emerged as an innovative technology in analytical chemistry, with the promise of fast analysis for a broad range of substrates. The column in LPGC-MS combines a wide analytical column and a narrow uncoated restrictor, resulting in higher flow rates, faster separation, and shorter analysis time. It has been used in analyzing nearly 30 kinds of pesticides in fruits, which can be separated and detected in less than 20 min (25). Indeed, it is important to explore and apply the LPGC-MS technology in other areas.

In this study, we revealed the degradation ability of *Rhodococcus erythropolis* XP on medium- and long-chain alkanes, including branch-chain alkane (pristane), and employed a novel LPGC-MS method (less than 12 min) to rapidly assess bioremediation efficiency. *Rhodococcus erythropolis* XP was proven to have potential in treating practical pollution by simulated bioremediation in oil sludge. Metabolic intermediates of both C16 and C20 degradation were determined, and their degradation pathways were proposed. Finally, a novel Baeyer-Villiger monooxygenase was cloned and characterized.

## MATERIALS AND METHODS

### Chemicals and medium

*n-*Dotriacontane (C32, ≥98%), pristane (98%), 2-hexadecanone (98%), and decyl acetate (98%) were purchased from Meryer (Shanghai) Biochemical Technology Co., Ltd. *n-*Icosane (C20, ≥99%), *n-*tetracosane (C24, ≥99%), *n-*octacosane (C28, ≥97%), *n-*hexatriacontane (C36, 96%), 1-hexadecanol (≥99%), 2-hexadecanol (95%), 1-icosanol (≥95%), 2-dodecanone (≥98%), ethyl acetate (≥99.9%), and alkane mixture standard (C7–C40) were obtained from Shanghai Aladdin Bio-Chemical Technology Co., Ltd. *n-*Hexadecane (C16, 98%) was obtained from Sinopharm Chemical Reagent Co., Ltd. Benzylacetone (98%), hexadecanal (98%), 2-heneicosanone (95%), and icosanal (98%) were purchased from Kaimeike (Shanghai) Pharmaceutical Technology Co., Ltd. Myristyl acetate (97%) was obtained from Shanghai Maikelin Biochemical Technology Co., Ltd. Progesterone (10 mM in dimethyl sulfoxide) and testosterone acetate were obtained from TargetMol Chemicals Inc.

*Rhodococcus erythropolis* XP was cultivated in basal salts medium (BSM) according to a previous study (26). Luria-Bertani medium (LB) per liter is composed of 10 g tryptone, 10 g NaCl, and 5 g yeast extract. Phosphate buffer saline (PBS) per liter contains 8.000 g NaCl, 0.245 g K_2_HPO_4_, 0.200 g KCl, and 1.440 g Na_2_HPO_4_.

### Growth and utilization of various long-chain alkanes

*Rhodococcus erythropolis* XP was previously isolated and identified (26). Cells grown to the late logarithmic phase in LB were collected at 8,000 rpm and washed three times, then suspended in sterile BSM. The resulting suspension was starved for 3 h and then incubated with different long-chain alkanes for adaptive growth. The adapted seeds were transferred (3% [vol/vol]) into 50 mL flasks containing 10 mL BSM with different concentrations of alkanes as the sole carbon source at 25°C, 200 rpm. For the culture supplemented with liquid alkanes (C16 and pristane), the formation of cell aggregation and their adhesion to the flask wall made it difficult to collect for determining OD_620 nm_, total protein, or dry weight. Photos were taken to measure the condition of bio-floccules. For solid alkanes (C20, C24, C28, C32, and C36), cell growth was monitored by measuring OD_620 nm_. Residual hydrocarbon was tested by extracting the growth medium with the same volume of hexane for GC-MS analysis. All tests obtained three biologically independent samples in this study.

GC-MS (Agilent and GC-7890B; MS-5977B) was equipped with a 50 m DB-5 column (J&M Scientific Flosom, CA) with helium as the carrier gas. The temperatures of the injector and transfer line were adjusted to 280°C and 300°C. The oven temperature program for analysis of C16, pristane, C20, C24, and C28 started at 70°C and was increased at a rate of 15°C/min to 240°C, then 20°C/min to 300°C and held for 5 min. The temperature for detecting C32 and C36 was set at 50°C for 2 min and ramped up to 300°C at a rate of 6°C/min, which was then kept for 16 min.

### Bioremediation of oily sludge

Oily sludge was collected from Daqing oilfield in Heilongjiang Province, China (46.58° N, 125.03° E). The sludge was thoroughly mixed and stored at 4°C after sampling. *Rhodococcus erythropolis* XP cultivated in LB medium was initially washed and resuspended in sterile PBS buffer (OD_620 nm_ = 10), then inoculated into the oily sludge (~10 g), while sterile PBS buffer was added in the sludge as a control. After mixing, samples were placed in an incubator at room temperature (25°C) for 21 days, and sterile PBS buffer was added every day to all samples to maintain humidity. Triplicate independent samples were collected by vacuum freeze-drying pretreatment, extracted by 20 mL acetone-hexane (1:1), and purified by solid phase extraction (SPME). Extraction was then analyzed by LPGC-MS, which was performed on Thermo Scientific TRACE 1610 GC coupled with TSQ 9610 (parameters were shown in Tables 2 and 3). A standard sample of alkane mixture (C7–C20, 0.6–25.0 ppm) was used to build the standard curve and calculate the recovery rate. Relative SD was obtained by calculating eight replicates of the standard sample (1.0 ppm). The alkane concentration in oily sludge was calculated using the following formula:


$$alkane\;concentration\;\left(mg/kg\right)=\frac{\rho \;\cdot \;V}{m\;\cdot \;\varepsilon }$$

**TABLE 2:** Parameters of TRACE 1610 for LPGC-MS

Program	Parameter
Sample introduction mode	Splitless
Injection temperature	280°C
Injection volume	1 µL
Column	15 m × 0.53 mm I.D., 1.0 µm film thickness TG-5 column with a 5 m × 0.18 mm I.D. non-coated restriction column at the inlet end
Carrier gas	Helium
Column flow rate	1.6 mL/min
Oven temperature program	50°C (held 2 min), ramped to 340°C at 50°C/min (held 5 min)

**TABLE 3:** Parameters of TSQ 9610 for LPGC-MS

Program	Parameter
Solvent delay	2 min
Ionization	EI
Analysis mode	Full scan
Source temperature	320°C
Transfer line temperature	300°C

ρ represents the concentration of alkanes calculated by the standard curve, mg/L. V represents the extracted volume, mL. m represents the dry weight of oily sludge, g. ε represents the recovery rate.

Microbial diversity analysis was performed by Shanghai Personal Biotechnology Co., Ltd. Total genomic DNA samples were extracted using the OMEGA Soil DNA Kit (M5636-02; Omega Bio-Tek, Norcross, GA, USA). The V3–V4 regions of bacterial 16S rDNA were amplified by primer 338F (5′-ACTCCTACGGGAGGCAGCA-3′) and 806R (5′-GGACTACHVGGGTWTCTAAT-3′). Sequence analysis was performed with QIIME 2 2019.4 (27) with slight modification. Briefly, raw sequence data were demultiplexed using the demux plugin followed by primers cutting with cutadapt plugin (28). Sequences were then quality filtered, denoised, merged, and chimera removed using the DADA2 plugin (29). No*n-*singleton amplicon sequence variants (ASVs) were aligned with mafft (30) and used to construct a phylogeny with fasttree2 (31). Taxonomy was assigned to ASVs using the classify-sklearn naïve Bayes taxonomy classifier in feature-classifier plugin (32) against the SILVA Release 132 Database.

### Determination of alkane metabolic pathways

To investigate the catabolic pathway of alkane degradation, C16 and C20 were used as the sole carbon source in BSM medium to identify potential intermediates, while sodium acetate was used as a control. To further illustrate the subterminal pathway of alkane degradation, 2-hexadecanol was used as the sole carbon source. *Rhodococcus erythropolis* XP was incubated with 250 mg/L of each substrate at 25°C, 200 rpm, for 12 h. Liquid cultures were extracted with an equal volume of ether at pH 12 and concentrated. After derivation with *N,O*-bis(trimethylsilyl)trifluoroacetamide at 70°C for 30 min, extracts were similarly analyzed by GC-MS.

### Phylogenetic analysis

Using the NCBI blastn search tool, BVMO_4041 residuals were compared to the reference sequences in the GenBank databases. Representative full-length sequences of BVMO were chosen. Phylogenetic analysis was performed based on the sequence alignment and truncation to the same length in MEGA version 4.0. ESPript 3.0 (https://espript.ibcp.fr) was used to render sequence similarity and secondary structure information (33). Maximum likelihood tree was calculated with the LG + G model using bootstrap values based on 1,000 replications.

### Cloning and expression of the BVMO_4041 in *E. coli* BL21(DE3)

Primer 4041F (5′-ACCATGGGCAGCAGCATGAGTCTTCCAGTTACAGATACCTCAGCAC-3′) and 4041R (TCCTTTCGGGCTTTGTCATTGTGCTACTGCCTTCTCGTCG) were used to amplify gene *BVMO_4041* of *Rhodococcus erythropolis* XP, and the linear vector of pET28a(+) was amplified by primer T7-F1 (GCTGCTGCCCATGGTATATCTCC) and T7-R1 (CAAAGCCCGAAAGGAAGCTGAG) for gene insertion. The plasmid was transformed into *E. coli* BL21(DE3) for protein expression. To verify the expression of this protein, *E. coli* BL21(DE3) carrying pET28a(+) with gene *BVMO_4041* was grown in 200 mL LB at 37°C, 200 rpm, until OD_600 nm_ reached 0.8, and then it was induced with 0.4 mM isopropyl-beta-D-thiogalactopyranoside (IPTG) at 15°C for 16 h. Cells were collected at 4,500 rpm for 10 min and resuspended in 10 mL PBS buffer. The resulting suspension was disrupted by ultrasonic breaking (60% of the maximum power), and the sample was analyzed using SDS-PAGE, while *E. coli* carrying empty pET28a(+) was used as a control.

### Degradation of ketone compounds by BVMO_4041

Engineered *E. coli* BL21(DE3) was cultured and induced in 1 L LB described in “Cloning and expression of the BVMO_4041 in *E. coli* BL21(DE3),” above. The strains were centrifuged at 4°C with 4,000 rpm for 20 min and resuspended with PBS buffer to adjust OD_600 nm_ to 10. The resulting suspension was starved at 25°C with 200 rpm for 3 h to get resting cells. Resting cells of 10 mL were incubated with 2-dodecanone, 2-hexadecanone, 2-heneicosanone, benzyl acetone, and progesterone, respectively, at 25°C with 200 rpm for 6 h. Productions were extracted by ethyl acetate at 25°C with 200 rpm for 20 min and detected by GC-MS as described in “Growth and utilization of various long-chain alkanes,” above.

## RESULTS

### Growth and utilization of medium- and long-chain alkanes

The alkane-degrading capacity of *Rhodococcus erythropolis* XP was determined by monitoring the cell growth and substrate degradation in BSM supplemented with different concentrations of alkanes (C16–C36 and pristane). In BSM with medium-chain alkanes (C16 and pristane), aggregation of cells was observed after 12 h incubation (Fig. S1). Aggregates that formed in the C16 medium were loose in a floating state, but those in the culture of pristane appeared to be more compact. With higher concentrations of pristane, cells formed firmer bio-floccules after 24 h, and more aggregates were settled in 48 h. Followed by the formation of aggregates, *Rhodococcus erythropolis* XP completely removed 0.1% (vol/vol) C16 and more than 80% of 0.1% (vol/vol) pristane in 60 h at 25°C (Fig. 2A and B). However, the concentration of pristane did not significantly begin to decrease until 36 h.

**Fig 1:**
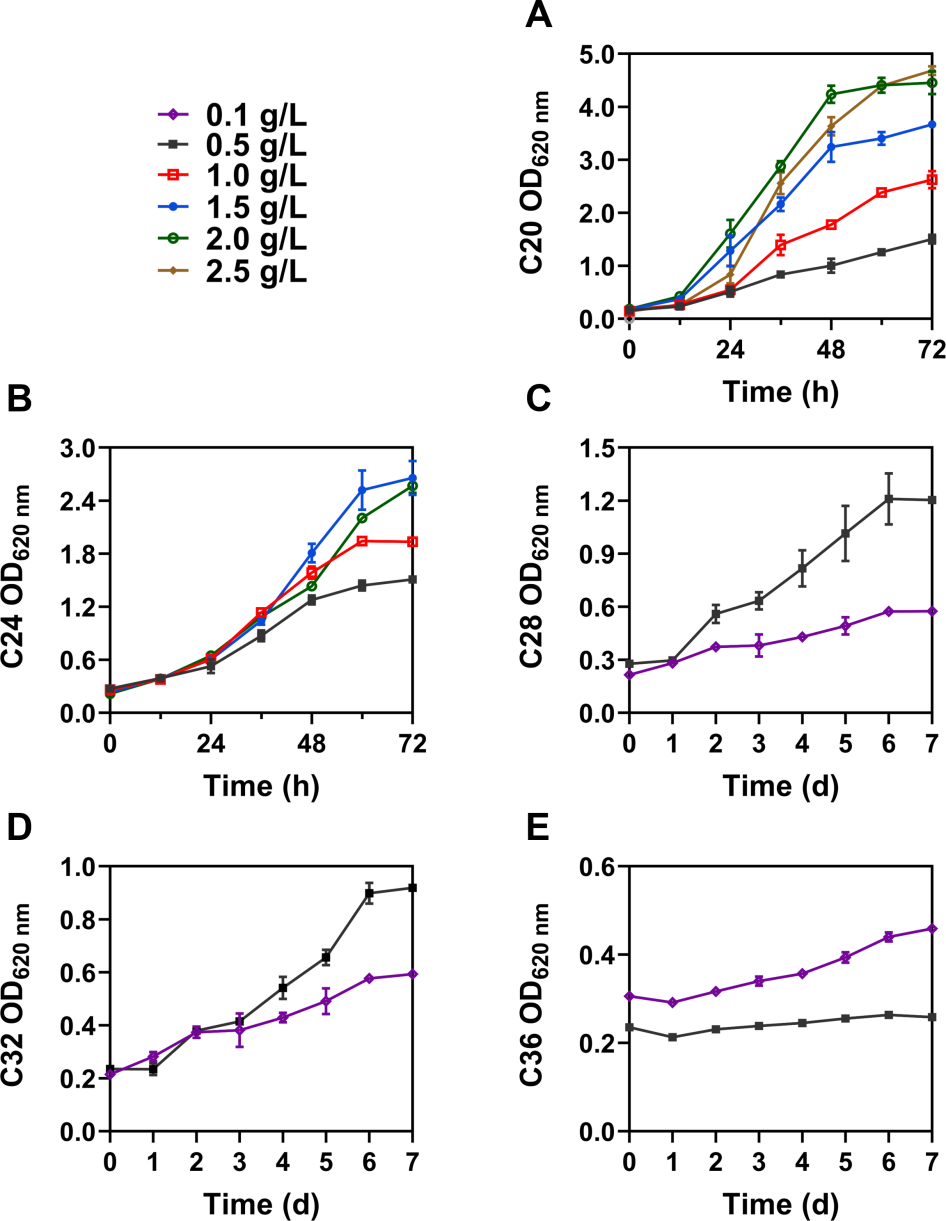
Growth curves of *Rhodococcus erythropolis* XP in BSM medium supplemented with long-chain alkanes with different concentrations. (**A**) *n-*Icosane. (**B**) *n-*Tetracosane. (**C**) *n-*Octacosane. (**D**) *n-*Dotriacontane. (**E**) *n-*Hexatriacontane.

**Fig 2:**
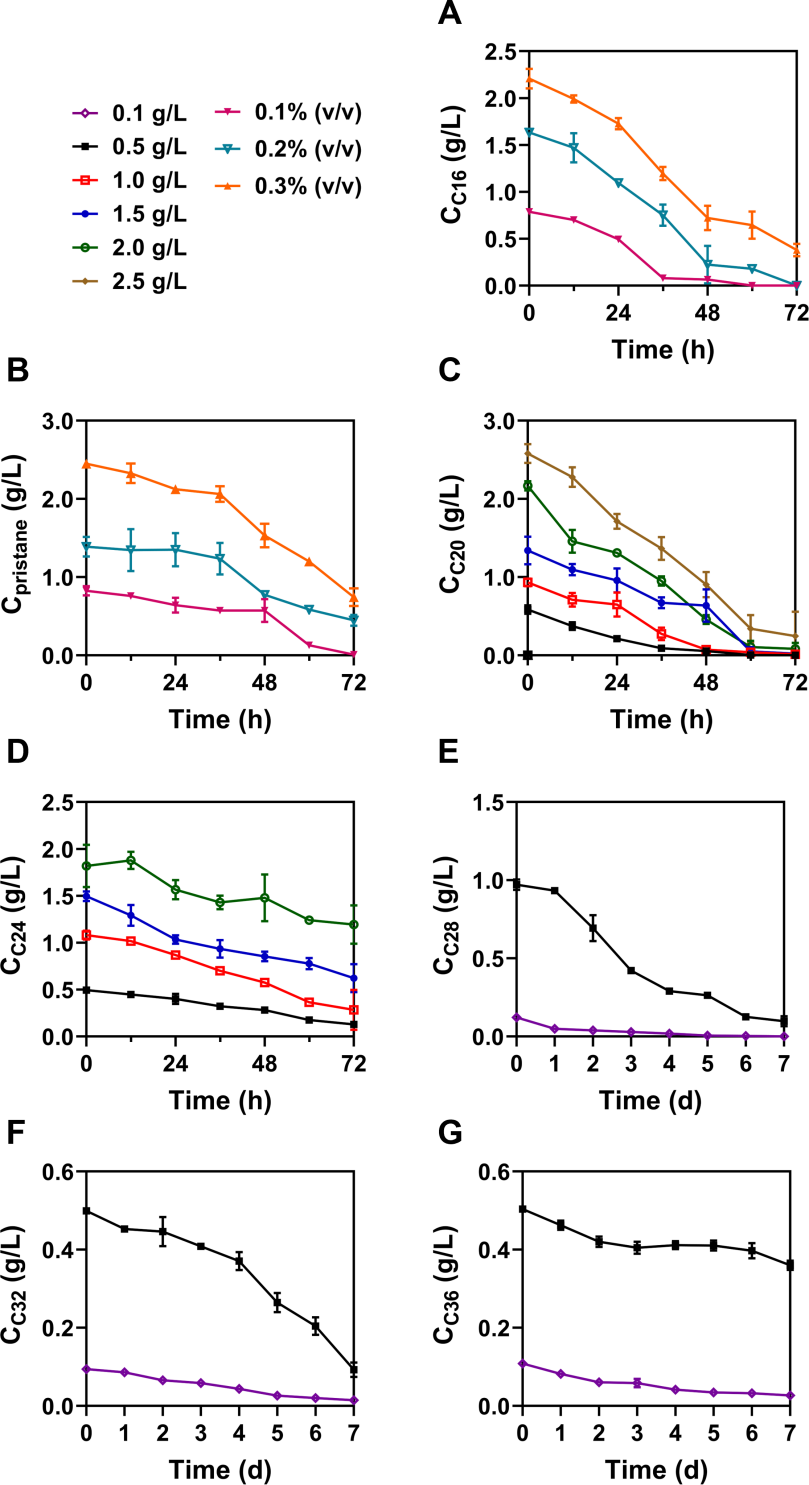
Degradation curves of *Rhodococcus erythropolis* XP in BSM medium supplemented with medium- and long-chain alkanes. (**A**) *n-*Hexadecane. (**B**) Pristane. (**C**) *n-*Icosane. (**D**) *n-*Tetracosane. (**E**) *n-*Octacosane. (**F**) *n-*Dotriacontane. (**G**) *n-*Hexatriacontane.

As shown in Fig. 2C, over 95% of C20 (500–2,500 mg/L) were degraded by *Rhodococcus erythropolis* XP in 72 h, and the OD_620 nm_ increased to 1.50–4.70 (Fig. 1A), suggesting a strong capacity to utilize the long-chain alkane C20. In BSM with less than 2,000 mg/L of C20, *Rhodococcus erythropolis* XP reached the exponential phase at about 12 h; however, it took approximately 24 h to get the same stage at 2,500 mg/L. This suggests that a higher concentration of C20 may require more time for cells to adapt and overcome any inhibitory effects of the substrate before entering the exponential growth phase. Furthermore, in the C24 medium (Fig. 1B and 2D), a delay in cell growth similarly occurred at 2,000 mg/L compared with 2,500 mg/L of C20. After 72 h, more than 70% of 500 mg/L C24 was degraded, and the OD_620 nm_ increased to 1.51. In the groups of C28, C32, and C36, *Rhodococcus erythropolis* XP grew more slowlier (Fig. 1E through G). After incubation of 7 days, OD_620 nm_ under each substrate of 500 mg/L reached 1.20, 0.92, and 0.27, respectively. In the groups of C28 and C32, biomass increased with increasing substrate concentrations, and *Rhodococcus erythropolis* XP utilized 500 mg/L of both C28 and C32 to over 80%. However, in the case of C36 at 500 mg/L, growth was stagnant compared to that at 100 mg/L, suggesting that high concentrations of C36 may have toxic effects on cells or interfere with their metabolic processes.

Of all the long-chain alkanes, *Rhodococcus erythropolis* XP exhibited the highest OD_620 nm_ value and degradation efficiency toward C20, suggesting that C20 may be the best available carbon source among long-chain alkanes. In the culture of C28–C36 *n-*alkanes, the delay in the growth of *Rhodococcus erythropolis* XP appeared to become longer as the chain length increased. The high hydrophobicity and low solubility of these alkanes make them less accessible to bacteria, thereby complicating their cellular uptake and metabolism.

### LPGC-MS application

**Fig 3:**
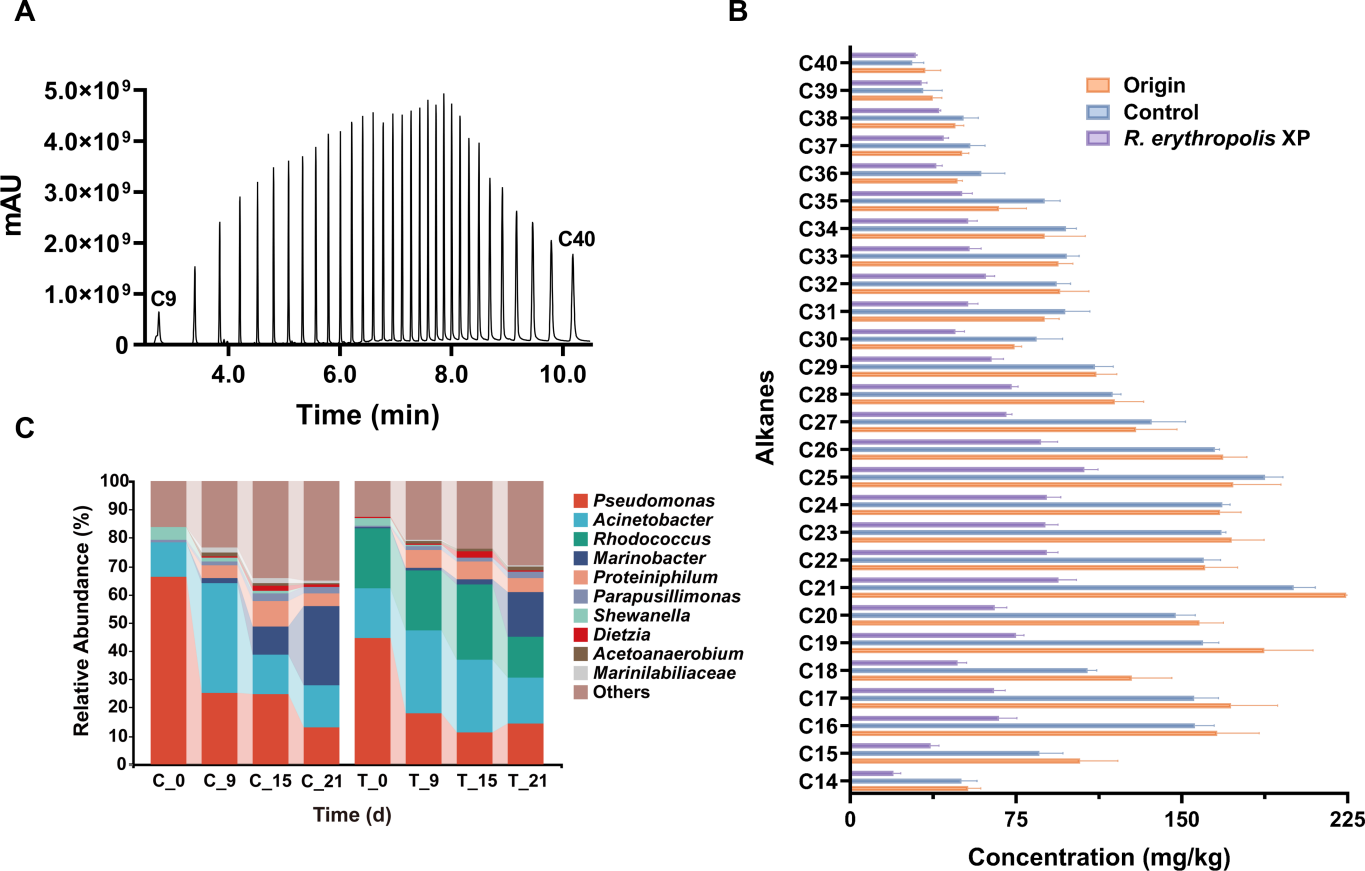
Bioremediation of oily sludge by *Rhodococcus erythropolis* XP. (**A**) Gas chromatograph in LPGC-MS of a mixed standard sample containing C9–C40 within 12 min. (**B**) Degradation of different alkanes in oily sludge by *Rhodococcus erythropolis* XP after 21 days. The control group was supplemented with PBS buffer. (**C**) The microbial composition in oily sludge at the genus level treated with *Rhodococcus erythropolis* XP (T group) or not (C group). Genera with relative abundance less than 0.03 were combined as others.

Considering the high volatility of short-chain alkane, different initial oven temperatures and ramping programs were tested in this study. The optimal starting point was settled as 50°C with a rate of 50°C/min to provide an increased speed in analysis, and the total ion chromatogram (TIC) of alkane standard mixtures was shown in Fig. 3A. C9–C40 *n-*alkanes can be successfully separated in 12 min. The repeatability assay (expressed as RSD, in %) was determined at concentration of 600 ppm and performed eight times (Fig. S2). The RSD values of the analytes were all less than 10% (C9–C40 *n-*alkanes). Calibration curves were constructed by alkane standard mixtures ranging from 600 ppb to 25 ppm, with correlation coefficients over 0.994. The recovery rate was determined on diatomaceous earth (free of alkane).

### Bioremediation of oily sludge

Oily sludge supplemented with PBS buffer (control group) and *Rhodococcus erythropolis* XP were respectively analyzed for microbial diversity (genus level) as well as concentrations of alkanes (Fig. 3B and C). The initial oily sludge predominantly contained C16–C26 alkanes. Following a 21-day incubation, notable biodegradation of C14–C30 alkanes was observed in sludge treated with *Rhodococcus erythropolis* XP, demonstrating its ability in bioremediation. As depicted in Fig. 3B, despite the lower concentration of long-chain alkanes (exceeding C35) compared to others, their decomposition proceeded at a slower speed, which may be due to their higher hydrophobicity. The microbial diversity data demonstrated that, in the original oily sludge, *Pseudomonas* (66.20%) and *Acinetobacter* (11.93%) were the dominant genera, which are commonly found in oil-contaminated sludge (34–36). Nine days later in the control group, *Acinetobacter* filled the niche with a quick increase in abundance to 38.79%, whereas *Pseudomonas* dropped to 25.09% and kept declining. However, after 15 days, the abundance of *Acinetobacter* decreased to 13.99%, while *Marinobacter* raised to 9.53% and topped the list at 21 days (28.06%). In the sludge inoculated with *Rhodococcus erythropolis* XP, *Pseudomonas* (44.63%), *Rhodococcus* (21.59%), and *Acinetobacter* (17.29%) were the dominant genera at the beginning. Similar to the results shown in the control group, at day 9, the abundance of *Pseudomonas* decreased (18.07%) and that of *Acinetobacter* increased (28.95%). However, *Rhodococcus* showed a relatively stable abundance during the first 9 days and then increased to 26.81% at day 15. Additionally, *Marinobacter*, initially present in low abundance (0.05%), also gradually increased to 1.96% at day 15. After 21 days, the abundance of *Pseudomonas* (14.16%), *Acinetobacter* (16.31%), *Rhodococcus* (14.74%), and *Marinobacter* (15.57%) was similar, reaching a relative balance. Indeed, *Rhodococcus* was well adapted to the oily sludge, capable of degrading alkane compounds with various carbon chain lengths.

### Metabolic pathway of saturated *n*-alkanes in *Rhodococcus erythropolis* XP

**Fig 4:**
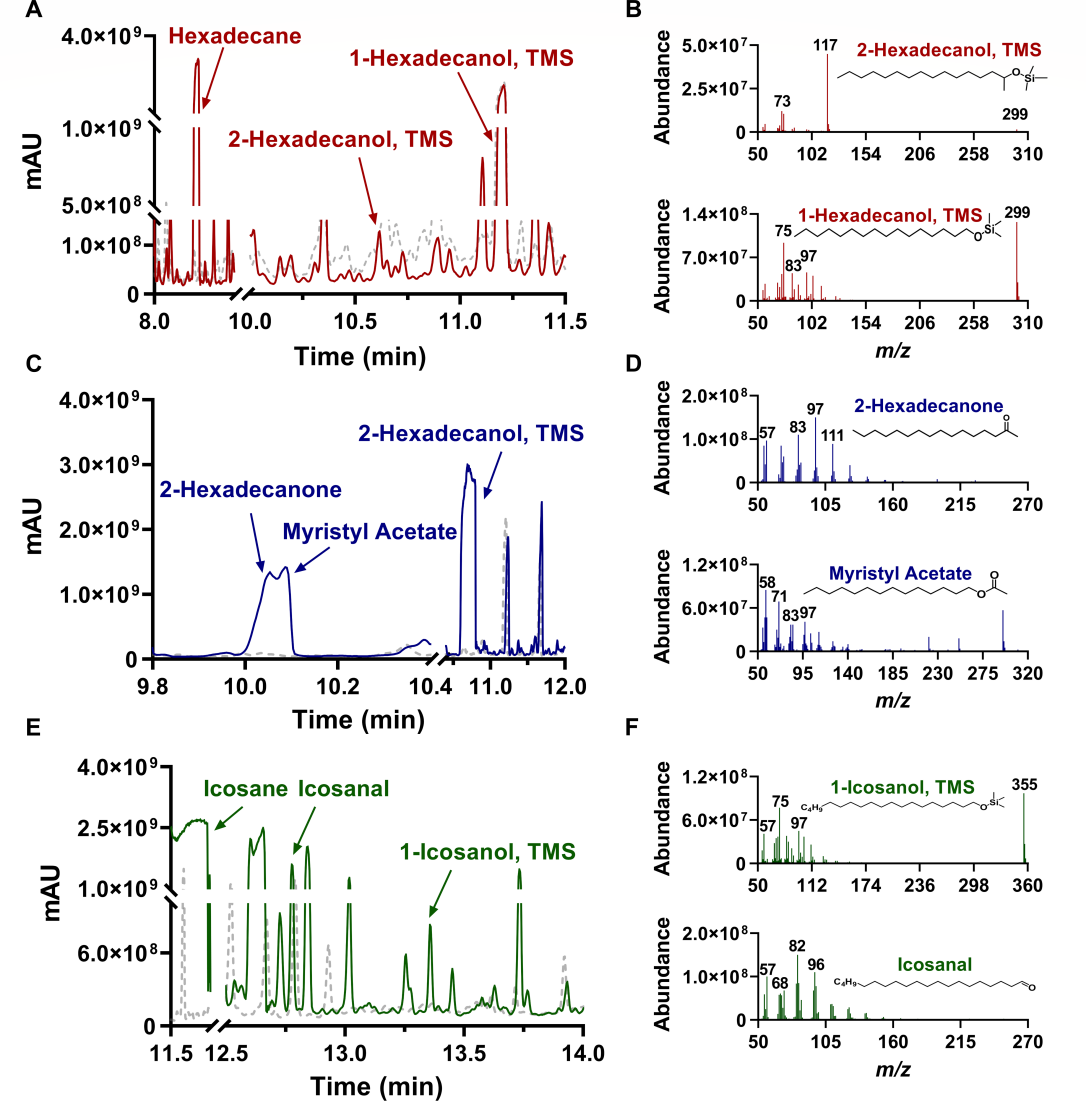
Identification of intermediate metabolites extracted from cultures supplemented with different substrates of *Rhodococcus erythropolis* XP using GC-MS. Cells cultivated on sodium acetate medium were used as a control, depicted with gray dash line. The gas chromatograms are on the left, and the mass spectra corresponding to the intermediate metabolites are on the right. (**A and B**) Intermediates extracted from the culture supplemented with hexadecane, red line. (**C and D**) 2-Hexadecanol, blue line. (**E and F**) Icosane, green line.

**Fig 5:**
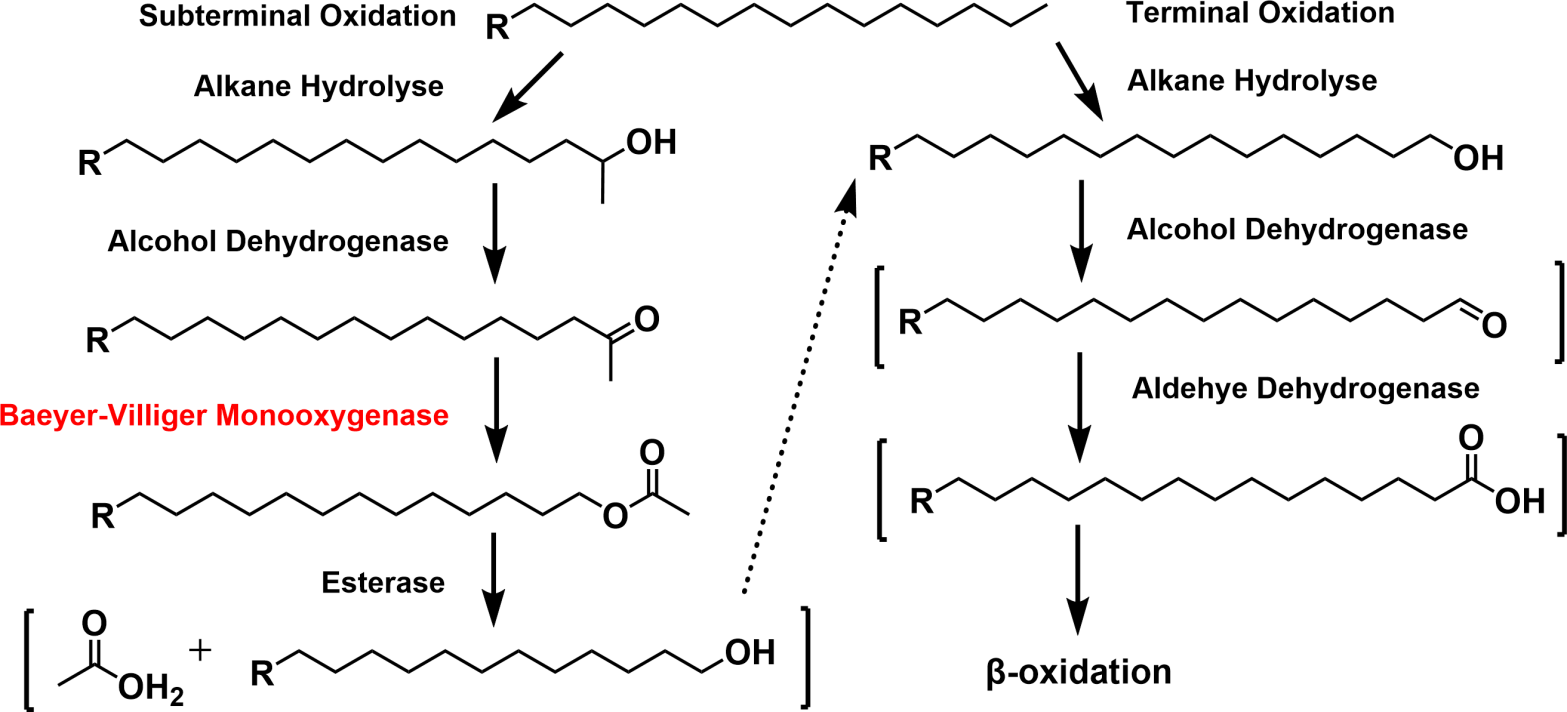
The presumed degradation pathway of hexadecane in *Rhodococcus erythropolis* XP according to the intermediate metabolites. Metabolites in square brackets are speculative and were not detected by GC-MS. The enzyme labeled in red was purified and verified its function, and the others in black were reported by previous studies that catalyze these steps.

Metabolites were extracted from cultures supplemented with hexadecane and icosane, respectively, and analyzed by GC-MS (Fig. 4). In the hexadecane culture, both 1-hexadecanol and 2-hexadecanol were identified by comparing the retention time and mass spectra with authentic standards, which indicated that *Rhodococcus erythropolis* XP oxidizes hexadecane via both terminal and subterminal pathways. Intermediates of *Rhodococcus erythropolis* XP grown on 2-hexadecanol as the sole carbon source were further examined independently. The further intermediates, 2-hexadecanone and myristyl acetate, in the subterminal pathway were identified as expected. The proposed degradation pathway of hexadecane was illustrated as Fig. 5. Nevertheless, in the icosane culture, only 1-icosanol was found, suggesting a different strategy of *Rhodococcus erythropolis* XP in degrading long-chain alkanes. These findings showed that both terminal and subterminal pathways coexist in *Rhodococcus erythropolis* XP and were associated with the chain lengths of substrates.

### Analysis of the Baeyer-Villiger monooxygenase

Since *Rhodococcus erythropolis* XP can degrade 2-hexadecanol via the subterminal alkane degradation pathway, it should carry the enzymes in this pathway. According to the whole genome, one putative gene, *BVMO_4041* encoding a BVMO was identified, which consists of 524 amino acids and has an estimated molecular weight of 57.6 kDa. A CLUSTALW alignment of BVMO_4041 and its orthologous protein was conducted to identify conserved regions and evaluate sequence similarity (Fig. S3). In previous studies, Type I Baeyer-Villiger Monooxygenases display a sequence fingerprint (FXGXXXHXXXW[P/D]) (37). The last amino acid in the fingerprint of BVMO_4041 was mutated to asparagine, and similar results were shown in Type I BVMOs of *Rhodococcus jostii* RHA1 (38). In addition, BVMO_4041 also contains two Rossmann fold domains (GXGXX[G/A]) to bind two cofactors (39, 40), and a particular region ([A/G]GXWXXXX[F/Y]P[G/M]XXXD) can be used to discriminate between Flavin monooxygenase and BVMO. The residues in this particular region were reported to support interaction with NADP^+^ coenzyme and binding of FAD cofactor (38).

**Fig 6:**
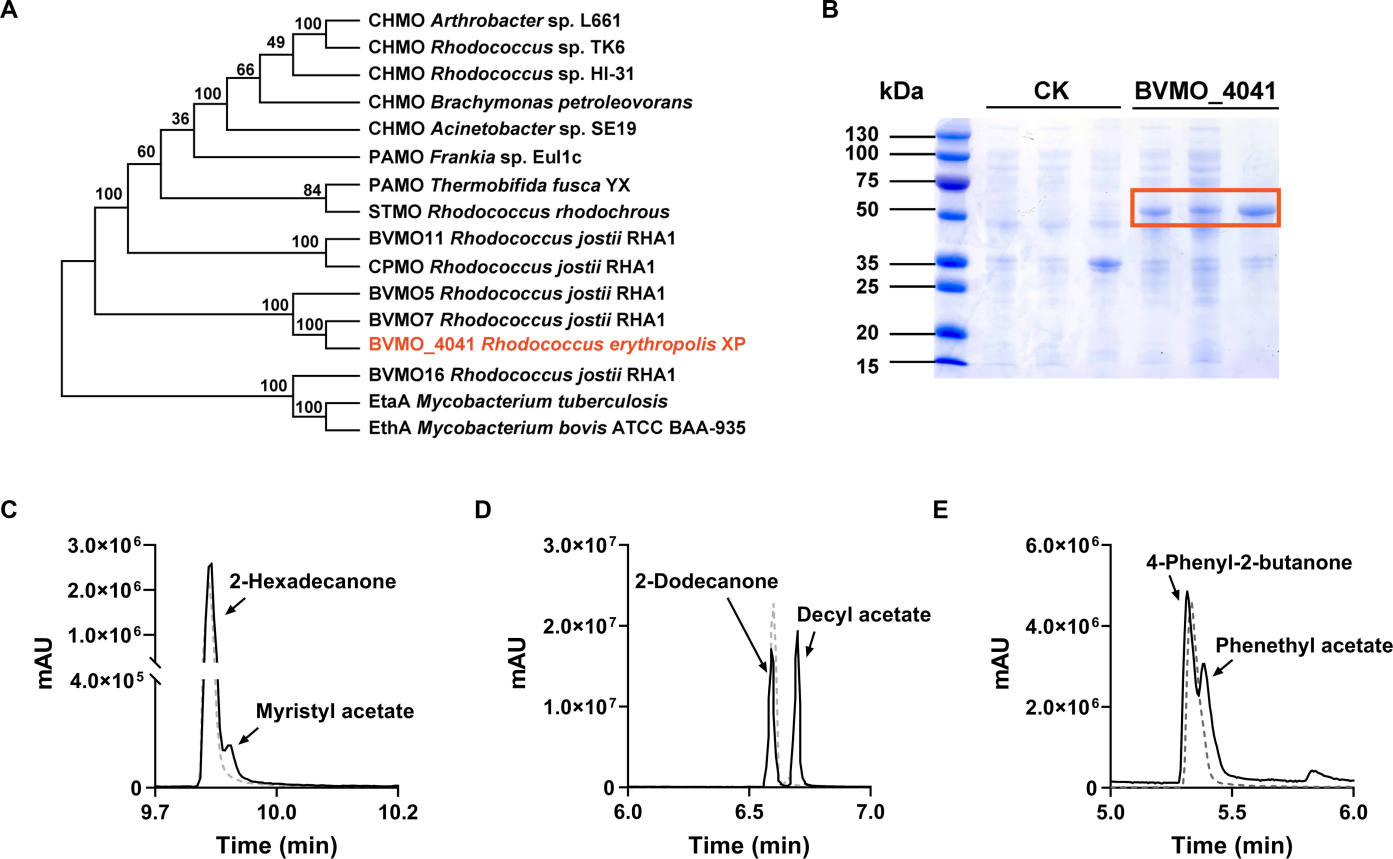
Characterization of Baeyer-Villiger monooxygenase (BVMO_4041). (**A**) Sequences of BVMOs with demonstrated activities were aligned, and the un-rooted phylogenetic trees were calculated by using CLUSTALW and maximum likelihood method. The numbers at the nodes are the bootstrap confidence values obtained after 1,000 replicates. The scale bar indicates distance in substitutions per nucleotide. (**B**) SDS-PAGE analysis of the expressed protein in control (empty vector) and experiment (BVMO_4041) group. From left to right, there are the whole-cell, supernatant, and pellet after ultrasonic crushing. (**C–E**) Gas chromatograms of extractions from cultures incubated with 2-hexadecanol (**C**), 2-dodecanone (**D**), and 4-phenyl-2-butanone (**E**) using resting cells (BVMO_4141 and an empty vector).

Compared with the proteins with known activity, BVMO_4041 shares a 43.67% sequence similarity with BVMO5 from *R. jostii* RHA1, 31.39% with steroid monooxygenase (STMO) from *Rhodococcus rhodochrous*, 29.70% with phenylacetone monooxygenase (PAMO) from *Thermobifida fusca* strain YX, and 83.40% with BVMO7 from *R. jostii* RHA1. The most similar enzyme, BVMO7, was an insoluble protein expressed by pET-YSBLIC-3C in *E. coli* (41). In another study, BVMO7 was expressed by the pCRE2 vector, and flavin reduction was observed. However, no enzymatic activity was detected among 39 substrates (38). A phylogenetic tree was constructed, followed by CLUSTALW alignment and cluster analysis, placing the BVMO_4041 of this study with BVMO7 and BVMO5 in a new cluster (Fig. 6A).

BVMO_4041 was cloned in pET28(a)+ and expressed in *E. coli* BL21(DE3) for the alkane degradation and other Baeyer-Villiger reaction assays (Fig. 6B). BVMO_4041 was found in both precipitate and supernatant, indicating a partial soluble form. Substrate specificity was tested with 2-heneicosanone, 2-hexadecanone, 2-dodecanone, progesterone, and phenylacetone in the resting-cell system (Table S1). When incubated with aliphatic methyl ketones, BVMO_4041 can oxidize 2-hexadecanone and 2-dodecanone into myristic acetate and decyl acetate, respectively (Fig. 6C and D). However, no activity was detected in the presence of 2-heneicosanone, implying that BVMO_4041 has a preference for different chain lengths of aliphatic methyl ketones. For progesterone and phenylacetone, which were the best substrates of STMO and PAMO, respectively, BVMO_4041 was unable to convert progesterone but displayed activity on 4-phenyl-2-butanone (Fig. 6E).

## DISCUSSION

*Rhodococcus erythropolis* XP is a highly efficient alkane degrader with a broad substrate spectrum, including C16–C36 *n-*alkanes and branch-chain alkanes. Most of the alkane-degrading strains can only mineralize a few types of the long- and branch-chain alkanes. Compared with *Pseudomonas aeruginosa* SJTD-1, *Rhodococcus erythropolis* XP can degrade not only C16–C24 *n*-alkanes but also C28–C36 *n-*alkanes (8). Regarding the same substrates, it could degrade them more efficiently. For instance, *Rhodococcus opacus* R7 consumed 51% of 1,000 mg/L C20 within 72 h, while *Rhodococcus erythropolis* XP degraded more than 90% of the same concentration in only 48 h (11). Moreover, it demonstrated a remarkable tolerance for higher concentrations of C20, degrading up to 95% of 2,500 mg/L C20 within 72 h. This high degradation efficiency positions C20 as a preferential carbon source for *Rhodococcus erythropolis* XP. In comparison with *Acinetobacter pittii* SW-1, which can degrade 500 mg/L C32 to over 30% within 7 days, *Rhodococcus erythropolis* XP consumed more than 80% of 500 mg/L C32 in the same period. In addition, a few strains were found to be capable of utilizing branch-chain alkanes. When 51% of 0.05% (vol/vol) pristane was used in 21 days by *Rhodococcus ruber* SBUG 82, *Rhodococcus erythropolis* XP utilized 80% of 0.1% (vol/vol) pristane in 60 h (14). Moreover, *Rhodococcus erythropolis* XP is also a promising option for oily sludge bioremediation. As we know, adding foreign bacteria for bioremediation usually faces the challenge of competing with indigenous bacteria (42). In *Rhodococcus erythropolis* XP treated oily sludge, the abundance of the *Rhodococcus* genus shows a slight increase at day 15, indicating an efficient utilization of alkanes for growth. However, we witnessed a decrease of *Rhodococcus erythropolis* XP abundance at the final phase of bioremediation, which suggested that the growth and development of *Rhodococcus erythropolis* XP may be inhibited. Meanwhile, using LPGC-MS accelerated the separation of long-chain alkanes; however, the retention time between branch- and straight-chain alkanes, which have similar boiling points, may become less pronounced, and peak broadening are often observed (data is not shown). To overcome this difficulty, paper layer chromatography of pretreatment of samples can be considered (43).

To decipher the degradation pathway of *Rhodococcus erythropolis* XP, the metabolites of C16 and C20 were characterized. Similar to previous findings (44), *Rhodococcus erythropolis* XP can both generate 1- and 2-hexadecanol under hexadecane, indicating the coexistence of terminal and subterminal oxidation pathways in *Rhodococcus erythropolis* XP. Possessing two alkane oxidation systems may enhance the bacterium’s capacity to adapt to complex environments by improving its carbon utilization efficiency (45). For the terminal oxidation pathway, it was found that the AlkB family of alkane hydroxylases, P450 monooxygenase, and long-chain alkane monooxygenase (LadA) can hydroxylate medium- and long-chain alkanes at the terminal position (23, 24). Although enzymes responsible for subterminal oxidation in medium- and long-chain alkane degradation have not yet been reported, engineering of a class II P450 enzyme (P450BM-3) from bacteria *B*. *megaterium*, which initially catalyzed fatty acid oxidation, exhibited a function of hydroxylating octane and other alkanes at the 2-, 3-, and 4- positions (46).

BVMOs have been widely investigated from a biocatalytic perspective, usually characterized with non-natural substrates for biotechnological applications. However, few studies focused on their physiological substrates, and their functional roles in bacteria remain unknown (47). We have demonstrated that *Rhodococcus erythropolis* XP could utilize medium-chain alkane by the subterminal pathway, and BVMO_4041 can convert medium-chain aliphatic ketone instead of long-chain aliphatic ketone. These results indicate that BVMO_4041 is involved in the subterminal pathway of medium-chain alkane metabolism of *Rhodococcus erythropolis* XP. Although BVMO_4041 shares high sequence similarity with BVMO5 (43.67%) and BVMO7 (83.40%) from *R. jostii* RHA1, it displayed differences in protein solubility and substrate preference, revealing it to be a novel Baeyer-Villiger monooxygenase. Compared to BVMO7 cloned in pET- and pCRE2-based expression system, BVMO_4041 was successfully expressed in a partially soluble form. Further experiments will need to elucidate the factors affecting protein folding between the two enzymes. As for substrate selectivity, both BVMO_4041 and BVMO5 could convert 2-dodecanone; nevertheless, BVMO5 did not function on phenylacetone, one of the common substrates for BVMO. Based on previous research, mutation of the M446 residue in the active site of PAMO reduced its affinity to phenylacetone. Aligned with the same active site, glycine in BVMO5 was found instead of methionine in BVMO_4041, which may indicate its deficiency of catalyzing phenylacetone. Also, similar results were found in PAMO and STMO, which shared over 50% sequence identity but showed different substrate specificity (48). Yet, it is hard to find a strict guideline between substrate specificity and the similarity of respective protein sequences (49). More substrates should be tested to investigate the preference of BVMO_4041. Meanwhile, it was notable that the reaction system of BVMO7 produced indigo blue, and some other constructs of BVMOs from *R. jostii* RHA1 exhibited an intense yellow color. This phenomenon was also observed in BVMO_4041 with a yellow-green color in the whole-cell suspension, which indicates a catalysis of indigenous chemicals in the medium or in the cells.

In conclusion, this study has successfully characterized a highly efficient alkane-degrading strain, *Rhodococcus erythropolis* XP. The degradation capabilities of alkanes and their pathway were demonstrated. Its application in bioremediation was further explored, along with the implementation of a novel GC-MS methodology. In addition, a new *BVMO_4041* gene encoding Baeyer-Villiger monooxygenase was found and analyzed. This paper provides valuable insights into *Rhodococcus erythropolis* XP as a promising candidate for both bioremediation efforts and microbial research.

## Data Availability

All data are available in the main text or the supplemental material. The materials and details that support the findings of this study are available from the corresponding author upon reasonable request. The data of genome sequencing can be found in the National Center for Biotechnology Information (NCBI) under accession number PRJNA1198704.

## SUPPLEMENTAL MATERIAL

Supplemental material aem.02124-25-s0001.docx Figures S1 to S3; Table S1.

ASM does not own the copyrights to Supplemental Material that may be linked to, or accessed through, an article. The authors have granted ASM a non-exclusive, world-wide license to publish the Supplemental Material files. Please contact the corresponding author directly for reuse.
