# Supplementary material for: Characterization of C16–C36 alkane degradation and oily sludge bioremediation by Rhodococcus erythropolis XP
Source: Appl Environ Microbiol. 2025 Dec 3;91(12):e02124-25. doi: 10.1128/aem.02124-25 (PMC12724338; doi:10.1128/aem.02124-25)
Supplement: Supplemental material — Figures S1 to S3; Table S1. [file aem.02124-25-s0001.docx]

**Supplementary Information:**

**Characterization of C16–C36 alkanes degradation and oily sludge bioremediation by** ***Rhodococcus erythropolis* XP**

Yan Zhang^1^†, Huan Liu^1^†, Shan Yu^2^, Ruocheng Pei^1^, Haiyang Hu^1^*, Weiwei Wang^1^, Ping Xu^1^, and Hongzhi Tang^1^*

^1^State Key Laboratory of Microbial Metabolism, and School of Life Sciences & Biotechnology, Shanghai Jiao Tong University, Shanghai 200240, People’s Republic of China

^2^Engineering Research Center of Agricultural Microbiology Technology, Ministry of Education & Heilongjiang Provincial Key Laboratory of Plant Genetic Engineering and Biological Fermentation Engineering for Cold Region & Key Laboratory of Microbiology, College of Heilongjiang Province & School of Life Sciences, Heilongjiang University, Harbin 150080, China

†These authors contributed equally to this study.

*Corresponding author: Prof. Hongzhi Tang or Dr. Haiyang Hu

Mailing address: State Key Laboratory of Microbial Metabolism, and School of Life Sciences & Biotechnology, Shanghai Jiao Tong University, Shanghai 200240, People’s Republic of China

E-mail: [tanghongzhi@sjtu.edu.cn](mailto:tanghongzhi@sjtu.edu.cn) or huhaiyang@sjtu.edu.cn

Tel: +86-21-34204066; Fax: +86-21-34206723


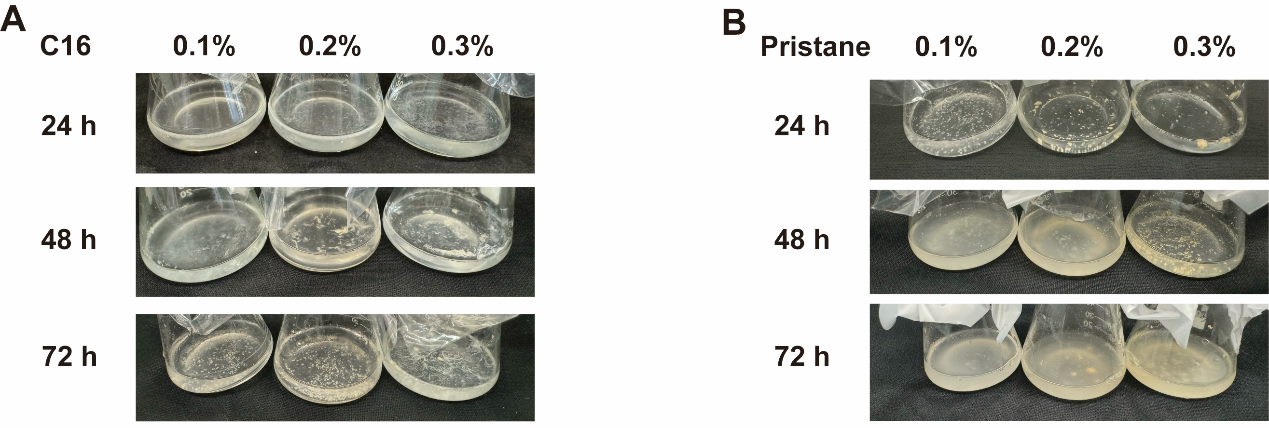


**Figure S1** Growth of *R. erythropolis* XP in BSM medium supplemented with C16 and pristane.


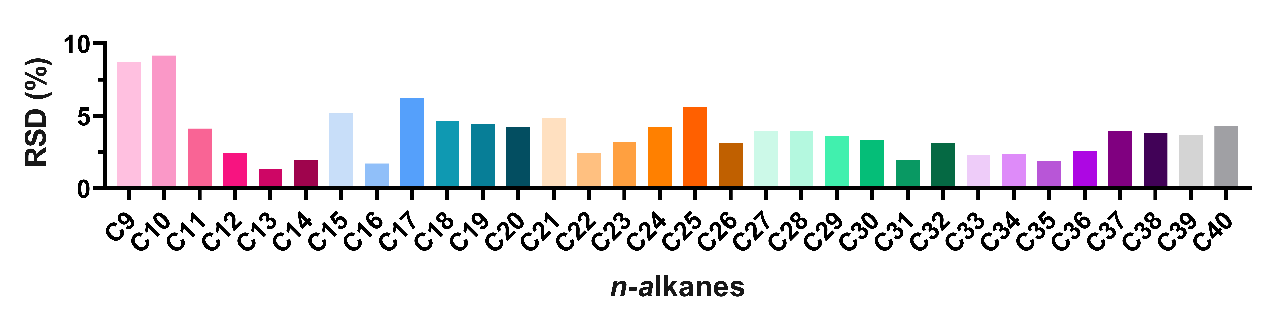


**Figure S2** Repeatability of rapid detection method of alkanes using Low Pressure Gas Chromatography-Mass Spectrometry (LPGC-MS) at 600 ppb concentration.


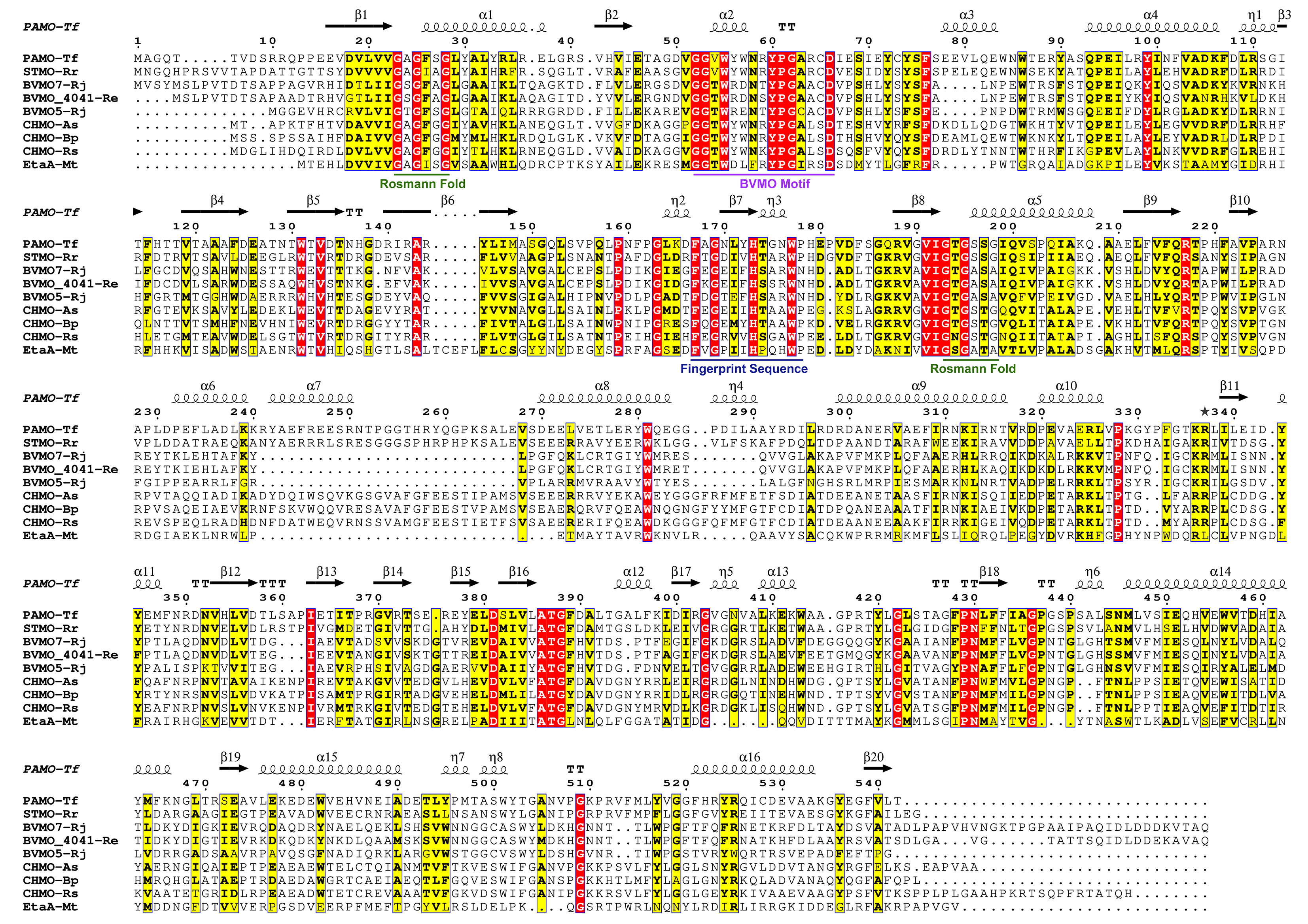


**Figure S3** Sequence alignment of different BVMOs. Strict identity residues are written in white characters (red box), and similar residues are written in black bold characters (yellow box). α-helices, 3_10_-helices and π-helices are displayed as medium, small and large squiggles respectively. β-strands are rendered as arrows, and strict β-turns as TT letters. PAMO-Ff = Phenylacetone monooxygenase from *Thermobifida fusca*; STMO-Rr = Steroid monooxygenase from *Rhodococcus rhodochrous*; BVMO7-Rj, BVMO5-Rj = Baeyer‐Villiger monoxygenases from *Rhodococcus jostii* RHA1; BVMO_4041 = Baeyer‐Villiger monoxygenase from *Rhodococcus erythropolis* XP; CHMO-As = Cyclohexanone monooxygenase from *Arthrobacter sp.* L661; CHMO-Bp = Cyclohexanone monooxygenase from *Brachymonas petroleovorans*; CHMO-Rs = *Rhodococcus sp.* HI-31; EtaA-Mt = Ethionamide‐activating monooxygenase from *Mycobacterium tuberculosis* H37Rv.

**Table S1** Summarization of the utilizable substrates for BVMO_4041.

|  | Natural substrates | | | Model sbustrates | |
| --- | --- | --- | --- | --- | --- |
|  | 2-heneicosanone | 2-hexadecanone | 2-dodecanone | progesterone | phenylacetone |
| Use | **-** | **+** | **+** | **-** | **+** |

“**+**” and “**-**” respectively represents that the substrates are available and unavailable.
